# Jasmonate Compounds and Their Derivatives in the Regulation of the Neoplastic Processes

**DOI:** 10.3390/molecules26102901

**Published:** 2021-05-13

**Authors:** Iwona Jarocka-Karpowicz, Agnieszka Markowska

**Affiliations:** Department of Analytical Chemistry, Medical University of Bialystok, Mickiewicza 2D, 15-222 Bialystok, Poland; agnieszka.markowska@umb.edu.pl

**Keywords:** jasmonic acid, anti-cancer drugs, structure–activity relationship, novel drug

## Abstract

Cancer is a serious problem in modern medicine, mainly due to the insufficient effectiveness of currently available therapies. There is a particular interest in compounds of natural origin, which can be used in the prophylaxis, as well as in the treatment and support of cancer treatment. One such compound is jasmonic acid (3-oxo-2-(pent-2’-enyl)cyclopentane acetic acid; isolated active form: *trans*-(-)-(3*R*,7*R*)- and *cis*-(+)-(3*R*,7*S*)-jasmonic acid) and its derivatives, which, due to their wide range of biological activities, are also proposed as potential therapeutic agents. Therefore, a review of literature data on the biological activity of jasmonates was prepared, with particular emphasis on the mechanisms of jasmonate action in neoplastic diseases. The anti-tumor activity of jasmonate compounds is based on altered cellular ATP levels; induction of re-differentiation through the action of Mitogen Activated Protein Kinases (MAPKs); the induction of the apoptosis by reactive oxygen species. Jasmonates can be used in anti-cancer therapy in combination with other known drugs, such as cisplatin, paclitaxel or doxorubicin, showing a synergistic effect. The structure–activity relationship of novel jasmonate derivatives with anti-tumor, anti-inflammatory and anti-aging effects is also shown.

## 1. Introduction

Neoplastic diseases constitute a major problem in modern medicine, mainly due to the insufficient effectiveness of currently available methods of anti-cancer therapy. Therefore, when analyzing the basic mechanisms regulating the development of neoplasms, new therapeutic solutions look for mechanisms that would prevent or extinguish the neoplastic process [1]. Particular attention is focused on compounds of natural origin that can be used both for the prevention and treatment of cancer. The National Cancer Institute has screened about 35,000 plant species that have potential anticancer activity, of which 3000 species have fully confirmed such activity [2]. Jasmonic acid and its derivatives are also among such compounds and show anti-cancer properties. However, while this action is not always effective enough for the needs of effective therapies, attempts are also made to combine jasmonic acid or its derivatives with other anti-cancer agents or radiotherapy, as well as chemically modifying the structure of these compounds in order to create derivatives with effective anti-cancer properties [3].

## 2. Jasmonic Acid and Its Derivatives Occurring in Plants

Jasmon compounds, called jasmonates, occur in almost all tissues of higher plants, i.e., flower plants, bryophytes and ferns, where they play the role of endogenous regulators of growth and development [4]. They are present in, among others, stems, roots, tubers, leaves, flowers, fruits and pollen. Jasmonic acid, the main representative of jasmon compounds, was first detected in the fungi *Lasiodiplodia theobromae*, and its methyl ester was isolated from the essential oils of the *Jasminum grandiflorum* olive family (now obtained commercially by synthesis) [5,6]. Jasmonians are found in over 160 plant families.

Jasmonic acid occurs in the green parts of plants in the form of conjugates with amino acids; in flowers, it is found with phenylalanine, tryptophan and tyrosine, in leaves with isoleucine or valine, and in fruits with isoleucine [7]. Moreover, jasmonic acid methyl ester is a component of essential oils and gives fragrance to many flowers (e.g., jasmine) and fruit (e.g., apple). Because of its fragrance properties, it is used by the perfume industry.

The content of jasmonates in plants is very diverse and ranges from 10 to 100 ng/g of fresh weight [5,8], depending on the type, species and age of the plant. It has been shown that there are more of these compounds in the generative parts of plants (pericarp, fruit and seeds) than in the vegetative parts (stems and leaves). Higher concentrations are also found in young plants as, with age, the jasmonates level decreases [5]. The increase in the content of jasmonates in the plant is also influenced by biological factors (insects and pathogens) and physicochemical factors (osmotic stress, drought, UV radiation, cooling and increased temperature, ozone) as well as mechanical damage (herbivores, mechanical stress) [8,9]. In plants, jasmonic acid has been found to be more frequently present than its methyl ester [2]. However, in the case of, for example, *Malus sylvestris* fruit, both compounds occur simultaneously. In recent years, these compounds have also been detected in lower plants (e.g., *algae*—*Chorella*) and fungi (*Gibberella fujikuroi and Botryodiplodia theobromae*). Unfortunately, previous studies have not shown in which specific parts of the plant they are synthesized.

## 3. Chemical Structure of Jasmonates

**Figure 1:**
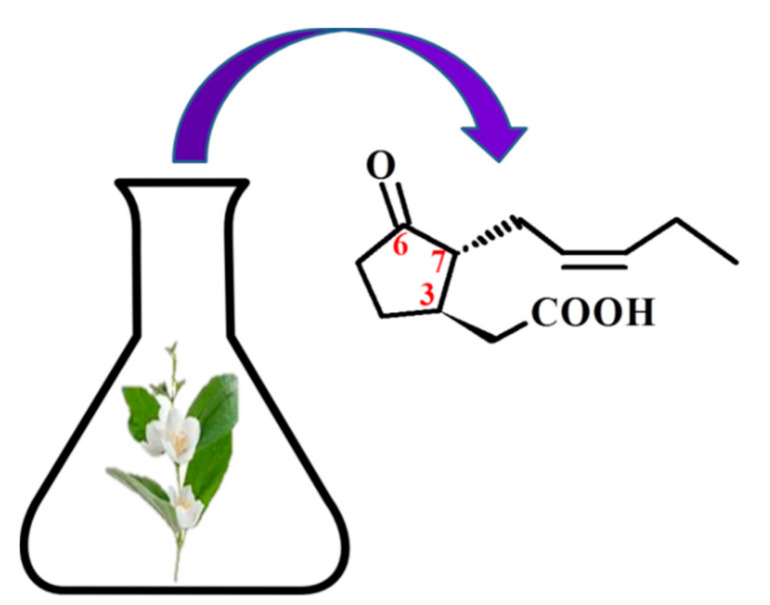
The structure of trans-(-)-(3R,7R)-jasmonic acid.

The basis of the structure of jasmonates is a cyclopentane ring with three different substituents in the C-3, C-6 and C-7 position, showing optical activity (Figure 1) [10]. The parent compound belonging to the jasmonate group is jasmonic acid 3-oxo-2-(pent-2’-enyl)cyclopentane acetic acid (IUPAC name), synthesized from linolenic acid found in the chloroplast membrane. The metabolites of jasmonic acid include various compounds obtained as a result of conjugation with isoleucine to obtain jasmonylisoleucine (JA-Ile) [9,11,12]; methylation to methyl jasmonate (MJ) (MJ) [9]; ester formation with glucose to 12-glucosyljasmonic acid [13]; decarboxylation to *cis*-jasmon [14] and hydroxylation to 12-hydroxyasmonic acid.

There are four known stereoisomers of jasmonic acid: *trans*-(−)-(3*R*,7*R*), abbreviated as (−)-JA; *trans*-(+)-(3*S*,7*S*) abbreviated as (+)-JA; *cis*-(−)-(3*S*,7*R*) abbreviated as (−)-*epi*-JA; *cis*-(+)-(3*R*,7*S*) abbreviated as (+)-*epi*-JA [15]. The naturally occurring jasmonic acid in plants is (−)-JA and (+)-*epi*-JA. Due to the fact that the *cis* stereoisomers are thermodynamically less stable, they epimerize at the C-7 atom to the stable *trans* form, which at the same time shows a higher biological activity. The biological activity of jasmonic acid has been found to be dependent on the presence of a carboxyl group at the C-1 position, a keto or hydroxyl group at the C-6 position, and a pentenyl side chain at the C-7 position [16,17,18]. Because of this structure, jasmonates inhibit, induce and/or stimulate changes that occur in plants at the morphological, physiological, cellular and molecular levels.

## 4. Pharmacological Activity of Jasmonates

The jasmonate family mainly consists of jasmonic acid, *cis*-jasmonate and methyl jasmonate (MJ), which are structurally similar to prostaglandins, especially those with anti-inflammatory action: prostaglandin D2 (PGD_2_) and 15-Deoxy-Δ12,14-prostaglandin J2 (15d -PGJ_2_) [19]. For this reason, many studies have been conducted to assess their effect on mammalian cells, which showed that jasmonates have a cytotoxic effect on neoplastic cells, while having no effect on healthy cells [20]. Jasmonates have been found to possess properties characteristic of anticancer drugs. Their action is characterized by high selectivity in relation to neoplastic cells, as well as effectiveness against neoplastic cells resistant to antineoplastic drugs. A cytotoxic effect of jasmonates was demonstrated in lymphoblastic leukemia cells (MOL-4) for the first time [21]. Further studies have also shown susceptibility to jasmonate in breast cancer cells (MCF-7), human melanoma cells (SK-28), androgen-responsive human prostate adenocarcinoma (LNCaP), cervix and murine lymphoma cells (EL-4) [19,22,23]. The greatest sensitivity to JA (increasing with increasing concentration, 0.5–3 mM) showed MOLT-4 cells, while SK-28, LNCaP and MCF-7 cells showed less sensitivity. MJ was more cytotoxic than jasmine acid. MJ induced 87.5% cytotoxicity in Molt-4 cells at a concentration of 0.5 mM. The same cytotoxicity was induced by JA at the highest used concentration [21]. The cytotoxic effect of MJ on cervical cancer cells was evaluated using a selected range of cervical carcinoma derived cell lines, including SiHa and CaSki (contain HPV16 DNA), HeLa (contain HPV18 DNA) and C33A (not containing HPV DNA). Cells were treated with 1–5 mM MJ for 24 h. C33A and CaSki cells were more sensitive to the MJ compared to HeLa and SiHa. The IC_50_ values were 1.7 mM for CaSki, 2.2 mM for C33A, 3 mM for HeLa and 3.3 mM for SiHa [24].

The antitumor activity of methyl jasmonate (MJ) was found to be higher than other jasmonates; therefore, MJ and its synthetic derivatives have recently been studied more intensively as promising compounds for the treatment of cancer [25]. MJ has been shown to prolong the survival of mice with EL-4 lymphoma and mice vaccinated with multiple myeloma cells (MM.1S) [21,26]. Survival rates were significantly higher in the group of lymphoma (EL-4) in mice treated with MJ (236 mg/kg) compared to untreated mice. Forty-five percent of the treated mice still live more than 5 months after the inoculation of the tumor cells [21]. In the case of murine multiple myeloma (MM) cell lines, MJ (0.5–2.5 mM by 24 h) significantly increased the survival rate. IC_50_ values for MJ were observed to be less than or equal to 1.5 mM for 15 of 16 (94%) cell lines. At the highest concentration tested, MJ caused more than a 90% reduction in the viability of all MM cell lines [26]. The cytotoxic effect of MJ (0.2–2000 µM) was also selective for MDA-MB-361 and T-47D human breast cancer cells [25]. The highest inhibition of the growth of T-47D and MDA-MB-361 cells was observed at 2 mM MJ (cell survival was 47% and 78%, respectively).

The preventive effect of MJ is primarily due to the ability to direct neoplastic cells to the path of apoptosis or necrosis. It is associated with the overproduction of reactive oxygen species (ROS) and the influence on the expression of proteins, such as p53, p21, and proteins from Bcl-2 and Bax families [24,27]. Western blot analyses indicated that MJ (2 mM, 5 and 24h incubation) induced a significant increase in p53 and decrease in p53 levels in CaSki cells. In HeLa and SiHa cells, a reduction in p53 and p21 levels was observed after 24 h of treatment [24]. In human non-small cell lung cancer cells (A549), jasmonate causes an increase in the expression of pro-apoptotic proteins from the Bcl-2 and Bax families [28,29]. MJ (2 mM) inhibits Bcl-2 protein expression in vitro in the case of prostate cancer cells (PC-3) [30]. The induction of apoptosis by methyl jasmonate is associated with decreased fluidity of tumor cell membranes and increased expression of tumor necrosis factor (TNFα) and its receptor 1 (TNFR1) in breast cancer cells (MDA-MB-435 and MCF7). The IC_50_ value of MJ for MDA-MB-435 cells was 1.9 mM and 2.0 mM for MCF-7 cells. The consequence of this action of jasmonate is the activation of caspase-8 in the external pro-apoptotic pathway. MDA-MB-435 and MCF-7 cells showed 35.0% and 37.2% apoptosis upon treatment MJ, respectively [31]. In contrast, MJ in human neuroblastoma cell lines caused decreased expression of XIAP protein and surwinin and MJ IC_50_ value for SK-N-SH and BE (2)-C cells were 1.39 and 1.35 mmol/L, respectively [32]. These results suggest that MJ may induce apoptosis through the activation of different signaling pathways in cervical cancer cells.

## 5. Antitumor Mechanisms of Action of Jasmonates Compounds

**Figure 2:**
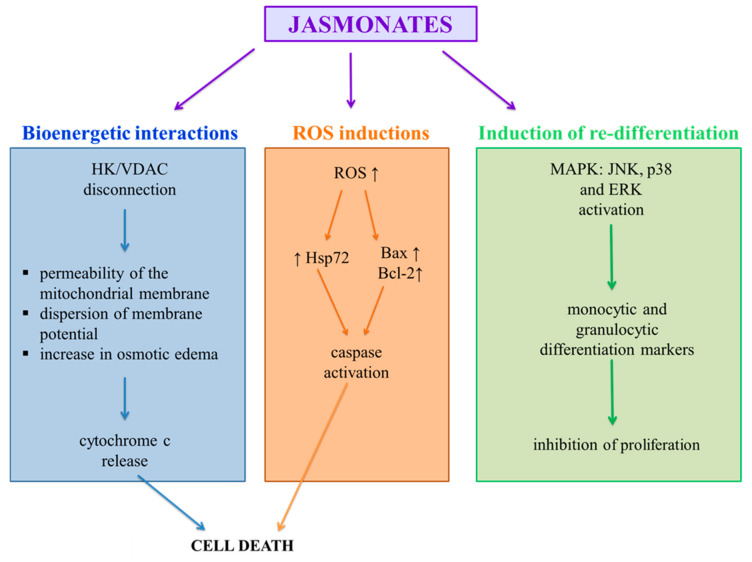
Antitumor mechanism of action of jasmonates. HK—hexokinases; VDAC—Voltage-dependent anion-selective channel 1; ROS—reactive oxygen species; MAPK—Mitogen Activated Protein Kinases; JNK—c-Jun N-terminal kinases; p38 protein; ERK—extracellular signal-regulated kinases; Hsp72—heat shock protein 72; Bax/Bcl-2—protein family.

Methyl jasmonate induces defense-related mechanisms in plants. Some aspects of the biosynthesis and function of jasmonate in plants resemble, in many respects, synthesis and function prostaglandins and leukotrienes in mammals (Figure 2). Because there is a certain balance between transforming healthy cells into cancerous cells and the inflammatory processes [33], the molecules structurally similar to natural anti-inflammatory compounds, especially low molecular weight molecules, such as jasmonates are considered as potential chemo-therapy agents. MJ has been shown to be active against cancer cells both in vitro and in vivo, without affecting normal cells. MJ may act as a chemosensitizer to some chemo-therapics, helping to overcome drug resistant.

### 5.1. Bioenergetic Interactions

Mitochondria, which provide cells with energy necessary for metabolic activities in the form of ATP, are extremely important for the functioning of eukaryotic cells. The action of cancer cell mitochondria differs from normal cells. They show a higher membrane potential, an increased rate of ATP production through glycolysis, and increased expression of proteins forming mitochondrial channels. Channel-forming proteins include VDAC proteins (voltage-dependent anion-selective channel 1) enabling the diffusion of ATP from the mitochondria into the cytoplasm [34]. Related to VDAC are the mitochondrial hexokinases (HK1 and HK2) responsible for the first step of glycolysis in the ATP production chain. Prolonged opening of VDAC channels by disconnecting the HK from VDAC causes a change in the permeability of the mitochondrial membrane, dispersion of membrane potential, an increase in osmotic edema and the release of pro-inflammatory factors, including cytochrome c, leading to cell death [22,27,35]. Because HK levels are up to 200 times higher in malignant tumor cells than in normal cells [36], this enzyme is an excellent molecular target for potential anticancer agents.

Methyl jasmonate, as a result of its interaction with hexakinase-2, reduces the binding of the enzyme to VDAC, which causes the disconnection of HK-2 from the mitochondrial membrane. The specific binding of jasmonate to HK2 was confirmed in an immunochemical test, using surface plasmon resonance and the study of VDAC activity of lipid bilayers without inhibiting kinase activity [37]. MJ has been shown to cleave HK1 and HK2 from VDAC in a dose-dependent manner in the mitochondrial fraction of CT-36 mouse colon carcinoma cells, MOLT-4 human leukemia, BCL1 mouse leukemia, B16 mouse melanoma and HCC hepatocellular carcinoma (LM3, BEL-7402, Hep3B, SMMC-7721). It was found that in the case of the action of MJ on cancer cells of neuroblastoma (SH-SY5Y) (IC_50_ = 1.65 mM after 24 h), lymphoma B cells (MJ; at 1 or 3 mM), and liver cancer cells (Hep3B) (IC_50_ = 2 mM after 24 h) the efficiency of glycolysis decreases and the level of cellular ATP is lower [38,39,40]. It has been observed that oligomycin (a mitochondrial ATP synthase inhibitor) does not increase the ATP depletion caused by MJ, whereas 2-deoxyglucose does [22,27,35].

### 5.2. Induction of Re-Differentiation

In healthy tissue, there is a balance between the proliferation, differentiation and death of cells. In cancer cells where proliferation is superior to death, differentiation is inhibited, resulting in the formation of poorly differentiated tissue [41]. Factors responsible for the regulation of cell proliferation and differentiation include cascading MAPKs: ERK (extracellular signal-regulated kinases), JNK (c-Jun N-terminal kinases), and p38 protein.

Methyl jasmonate has the ability to induce the MAPK pathway which determines the re-differentiation of neoplastic cells. Two independent processes were observed in MOLT-4 leukemic cells and A549 lung carcinoma treated with MJ (0.5 mM and 4.937 mM for 24 h, respectively): apoptotic cell death and the release of JNK and p38 proteins, causing activation of the transcription factor AP-1. Although AP-1 is a protein involved in both apoptosis and differentiation, no effect of AP-1 on the apoptotic pathway was found in MOLT-4 and A549 cells. In MCF-7 and MDA-MB-435 breast cancer cells (both receptor-dependent and receptor-independent), methyl jasmonates (2 mM for MCF-7 and 1.9 mM for MDA-MB-435) caused apoptosis in both cell types, but activated the p38 and ERK pathways through MAPK only in the receptor-independent cells [28,31].

MJ (0.4 mM for 24 h) activated the MAPK pathway in the human myelocytic leukemia cell line HL-60, which resulted in the differentiation of cells into monocyte-like granulocytes instead of apoptosis [42]. The differentiation effect was inhibited by PD98059, a MEK/ERK inhibitor, confirming the involvement of MJ in the activation of the MAPK/ERK dependent pathway [43]. In the same way, MJ also acts on U937 histiocytic lymphoma cells and THP-1 acute monocytic leukemia cells, which are model cells for studying monocyte behavior and differentiation [35].

Acute myeloid leukemia (AML) is one of the most severe forms of leukemia because it is largely resistant to chemotherapy. It has been shown that MJ stops the growth of leukemic blasts and stimulates their differentiation into normal cells, as confirmed by the expression of differentiation markers, such as reduction in NBT nitro-tetrazolium blue (for myelomonocytes differentiation), morphological differentiation into granulocytes and the expression of antigens of cell differentiation CD14 (specific for monocytes) and CD15 (specific for granulocytes) (IC_50_ = 347 µM) [41]. MJ (0.4 mM for 24 h) also induced the expression of the calcium-binding protein S100P in the AGE/RAGE glycation pathway, leading to an increase in MAPK activity [44].

Moreover, MJ (IC_50_ = 347 µM) induces the transcription of the C/EBP protein binding the regulatory DNA sequences of CCAAT, which may contribute to the initiation of granulocyte differentiation in human myeloid leukemia cells [35,41].

### 5.3. Induction of Apoptosis by ROS

Excessive production of ROS in cancer cells can lead to the oxidative modification of DNA and lipids, which in turn causes damage to cell membranes. Both jasmonic acid and its methyl ester cause a significant increase in ROS levels in glioblastoma cells (C6), non-small cell lung cancer cells (A549 and H520) and in uterine cancer cell lines (HeLa and CaSki) [28,45,46,47,48].

In human non-small lung cancer cells (A549), MJ (A549—5 Mm; PC-3—2 mM) increases the expression of pro-apoptotic proteins from the Bcl-2, Bcl-Bax and Xs families [28], and in PC-3 prostate cancer cells, an increase the expression of the anti-apoptotic protein Bcl-2 [30]. This results in cell cycle arrest in the G2-M phase and activation of executor caspase, followed by directing cells to apoptosis. The suppression of MJ-induced apoptosis by antioxidants, such as N-acetylcysteine and catalase (specific H_2_O_2_ inhibitors), but not by inhibitors of hydroxyl radicals and superoxide ions, suggests that hydrogen peroxide is one of the important factors associated with MJ-induced anti-cancer effects [22,48,49]. Intracellular H_2_O_2_ and O^2−^, and mitochondrial ROS were prominently increased in response to 5 mM MeJA in C6 cells [47].

In addition, methyl jasmonate has been shown to reduce the levels of survivin, a protein involved in the inhibition of apoptosis, in uterine cancer cell lines (HeLa and CaSki) and to induce heat shock protein 72 (Hsp72) in C6 glioma cells [22,48]. MJ also leads to an increase in the sensitivity of cells to ionizing radiation [50]. On the other hand, in esophageal squamous cell carcinoma (KY170R) cells, MJ (200 µM for 24 h) increases the level of ROS and inhibits the activity of 11-ketoprostaglandin reductase (by roughly 30%), which dramatically increases cell death in response to X-rays [46].

## 6. Therapeutic Benefits of Combining Jasmonates with Anti-Cancer Drugs

In addition to the fact that jasmonates induce apoptosis in tumor cells, they can also be combined with other anti-tumor agents to achieve synergistic anti-tumor effects. In fact, many modern chemotherapy procedures use multicomponent combinations of drugs that allow for lower doses to be administered, can reduce undesirable side effects and even overcome drug resistance [20]. Therefore, studies have been carried out to evaluate the combination of the effects of MJ and various other anticancer agents [51] that are routinely used in clinical practice: BCNU (carmustine), cisplatin, paclitaxel (taxol) [52] and doxorubicin (adriamycin) or 3-bromopyruvate (3-BrP) (Table 1).

The interactions of these combination drugs have been observed in many cell lines of malignant tumors such as: breast, lung, prostate and pancreatic cancer as well as leukemia [53], where MJ drastically reduced the IC_50_ values of the used chemotherapeutic drugs, while reducing of side effects of these drugs. The combination of MJ (0.1 nM) and BCNU (carmustine) therapy (1, 10 and 25 µg/mL—PaCa-2 cell; 2.5 and 5 µg/mL—BCL1 cell) had an adverse effect on pancreatic cancer cells (PaCa-2; BCL1) causing their apoptosis, which was not observed with BCNU alone. It follows that the influence of BCNU on mitochondria [54] makes them hypersensitive to MJ, resulting in over-additive cytotoxic effects. Another study also showed a positive effect of MJ together with perillyl alcohol (POH), which increased the cytotoxicity of cisplatin in breast cancer cells (MDA-MB-231, MDA-MB-435, MCF7) [55].

The combination of MJ (0.5–3 mM) and 2DG (2-deoxyglucose, glycolysis inhibitor) (1 and 2 mM) also resulted in a synergistic cytotoxic effect on tumor cells (sarcoma SaOS-2; MCA-105), possibly due to the interaction of both MJ-induced oxidative phosphorylation of ATP biosynthesis and 2DG-induced ATP glycolysis [22,56]. Most importantly, in vivo experiments have shown that the combination of MJ and doxorubicin has a synergistic effect on mouse leukemia (BCL1) [22]. Moreover, pre-incubation with MJ (0.5 mM), at non-cytotoxic concentrations, may sensitize colorectal cancer (CRC) cells to ligand-induced apoptosis, inducing TRAIL (tumor necrosis factor-related apoptosis-inducing ligand) (100–200 ng/mL), resulting in synergistic cell death through enhanced caspase activity [57,58].

TRAIL receptors are highly expressed in primary tumors and various tumor cell lines [59], which makes this pathway of cytotoxicity very specific for neoplastic cells while sparing most of the normal cells. Therefore, TRAIL-induced cell apoptosis is a very attractive potential target of anti-cancer therapy [57]. TRAIL, induced by MJ, mediates the reduction in survivin, a member of the Inhibitors of Apoptosis Proteins family (IAP). This study also shows that MJ, by inhibiting transcription, influenced the signal transduction pathways, resulting in a reduction in survivin mRNA [20]. Nevertheless, many tumor cells are intrinsically resistant to TRAIL-induced apoptosis. Therefore, the results of synergistic cell death achieved by the combination of MJ and TRAIL, through bypassing the TRAIL resistance barrier, have great potential in tumor therapy as both agents are highly selective for tumor cells.

Moreover, studies on prostate (PC-3) and breast cancer cell lines (MDA-MB-435) showed that treatment with jasmonates resulted in increased expression of TNFR1 and caspase-8 and caspase-3 activation [22], showing that jasmonates can act directly on the extrinsic apoptotic pathway in addition to intrinsic mitochondrial apoptotic pathway. Additionally, the IAP antagonist, an N-terminal peptide consisting of seven Smac residues (SmacN7), synergistically significantly increased MJ-induced cytotoxicity in human cancer cells, but not in normal epithelial cells, and acted simultaneously through caspase-9 dependent and independent pathways [60,61]. These findings suggest that inhibition of IAP may facilitate MJ-induced cytotoxicity and may be of advantageous value for the further development of jasmonate-based chemotherapy.

MJ (0.5–3 mM) also increased the effectiveness of therapy with the use of 3-BrP (12.5; 25; 50; 100; 200; 400 μM; IC_50_ value was 70 μM). This polytherapy was more effective than monotherapy with 3-BrP, MJ, and also surprisingly with cyclophosphamide as a routine treatment for breast cancer in tumor bearing mice, as observed by reducing tumor volume and increasing the percent inhibition of tumor growth. Moreover, the applied therapy had no appreciable side effects on the kidneys, liver, immune system and body weight [53].

Recently, MJ has also been shown to be effective in cooperating with cisplatin and radiotherapy in the treatment of cervical cancer cells by significantly reducing the doses of radiation and cisplatin required to inhibit these cells’ survival [22]. This study showed, for the first time, that alpha radiation selectively reduces cell viability and cervical cancer cell survival, and that alpha radiation also works with MJ in reducing cell viability, as shown in some of the cervical cancer cell lines used (SiHa, CaSki, HeLa and C33A) [62]. In addition, MJ can be administered along with conventional X-ray (0.25–3 Gy) and cisplatin therapies, increasing their cytotoxic efficacy while lowering the dose, avoiding possible side effects.

## 7. New Synthetic Derivatives of Jasmonates

One of the main problems with the use of natural jasmine compounds, such as methyl jasmonate as drugs, is the need to use high doses in millimolar concentrations, regardless of whether they relate to solutions in organic solvents or water. The necessity to use high concentrations results from the hydrophobic properties of jasmonate molecules and their low availability in the culture medium (in vitro) or in physiological fluids (in vivo). Therefore, there is growing interest in the use of novel jasmonic acid derivatives that reduce solubility limitations while having improved properties as potential therapeutics in relation to parent molecule (Table 2 and Table 3).

The basic modification changing the activity of jasmonates is the introduction of a double bond in the cyclopentyl ring in molecules of jasmonate. Chemically, α,β-unsaturated carbonyl compounds (enone) are electrophilic centers that are highly susceptible to addition reactions with nucleophiles, such as free sulfhydryl groups of reduced glutathione or cysteine residues in proteins. Prostaglandins PGA1, PGA_2_, PGJ_2_ and their metabolites 15-deoxy-12,14-PGJ_2_ (15d-PGJ_2_), D12-PGJ_2_ and D7-PGA1 are compounds in which cyclopentenone is a pharmacophore responsible for the biological activity of these compounds [63].

Therefore, the introduction of unsaturation bonds into the methyl jasmonate (MJ) molecule to give methyl 4,5-didehydrojasmonate (DHJM) resulted in the formation of a compound exhibiting a much lower concentration of antitumor activity similar to that of the naturally occurring MJ (Table 2 and Table 3). DHJM was effective in inhibiting the growth of HL-60 human myeloid leukemia cells (IC_50_ = 12 μM for DHJM vs. 347 μM for MJ, Table 1) and induced concentration-dependent MAPK activity (in the concentration 0.03–0.3 mM of DHJM vs. 0.3–1 mM of MJ for 3 h of the incubation) [41]. DHJM showed significant anti-inflammatory properties by dramatically suppressed of nitric oxide (NO), interleukin-6 (IL-6) and TNF-α in murine activated macrophages (RAW264.7) [64]. These actions have been shown to mediate the NF-κB pathway, by inhibition of the nuclear translocation of phospho-p65 protein and cytoplasmic degradation of IκB-α, resulting in decreased NF-κB transactivation and the reduction in miR-155 expression.

Other derivatives were obtained by introducing two atoms of bromine, chlorine or iodine in the side chain and two in the cyclopentane ring, of which the 5,7,9,10-tetrabromo derivative showed significant cytotoxicity against the following cell lines: B16-F10 melanoma (IC_50_ 0.042 mM), MOLT-4 human lymphoblastic leukemia (IC_50_ 0.009 mM), MCF7 human breast cancer (IC_50_ 0.015 mM), human pancreatic cancer MIA PaCa-2 (IC_50_ 0.09 mM) and D122 murine lung cancer (IC_50_ 0.25 mM), and effectively prevented B16-F10 cell adhesion and inhibited lung metastasis at a much lower dose than MJ (Table 2) [65]. Tetrabromoderivative MJ was the most spatially molecule of the five tested molecules in the paper. The authors suggest that large bromo groups with spatial hindrance induced steric effects giving a better match of the molecule to the putative binding site to the potential molecular target.

Further modifications to the jasmonate molecule included the introduction of various halogens into the α-cyclopentenone position while maintaining the double bond in the ring, and/or introduction of an amide residue instead of an ester (Table 3) [66]. The authors concluded that the presence of the cyclopentenone fragment is necessary to maintain the anti-inflammatory activity. The presence of halogens increases this activity even in the presence of saturated bonds in the side chain. The compounds showed no cytotoxicity but showed higher anti-inflammatory activity than natural prostaglandins in lipopolysaccharide-activated inflammation in RAW264.7 cells. The inhibitory activity of methyl 5-chloro-4,5-didehydrodihydro-jasmonate was assessed by their effect on the production of pro-inflammatory mediators (NO, IL-6, and TNF-a).

In 2012, Dang et al. synthesized a series of methyl jasmonate derivatives, tested their resistance to hydrolysis and converted them into derivatives with a chlorine atom in the α position of cyclopentenone (Table 2) [67]. The anti-inflammatory activity of a number of aryl, hydroxyalkyl, methoxyalkyl and branched alkyl esters was tested to examine their lipo/hydrophilicity and steric effect on activity. The most active analogs in this series were t-butyl (Tabel 3) and hydroxyethyl esters. It was confirmed that chain branching and increased hydrophilicity in relation to the methyl moiety in MJ affects biological activity.

The replacement of 1,2,4-oxodiazole in place of the methyl ester fragment of jasmonate (Table 2) and the reduction in the ketone in cyclopentanone to the hydroxyl group resulted in an active inhibitor of hexokinase II, enzyme present in excess in neoplastic cells (IC_50_ 0.29 μM), as well as demonstrated cytotoxicity to human cell lines: lung cancer A549 and SKOV-3 ovarian carcinoma (Table 2) [68].

During synthesis, the activities of the intermediate products were also investigated, but the focus was only on those that showed enzyme inhibitory activity only at values lower than 1 μM. So far, similar jasmonate compounds have not been described, and the idea was based on a moderate analogy to lonidamine (derivative of benzodiazole), a hexokinase II inhibitor, a candidate for clinical trials as an anti-cancer drug. Nevertheless, currently in therapy there are a few commercially available drugs containing 1,2,4-oxodiazole discussed fragment (*Oxolamine*, *Prenoxdiazine*, *cough suppressant; Butalamine*, *vasodilator; Fasiplon*, *nonbenzodiazepine anxiolytic drug; Pleconaril*, *antiviral drug and other*) [69].

Jasmonate derivatives (a methyl group in place of methylcarboxylic residue) con-taining heteroatoms in the cyclopentyl ring (N, O) in place of some carbon atoms have been tested for their odor properties (Table 3). The unsaturated cis double bond, present in the alkyl side chain at position 2 in natural cis-jasmine, plays a very important role in the fragrance properties, and the introduction of the double bond into the heterocyclic jasmine analogs, both *cis* and *trans*, leads to an increase in odor intensity in comparison to their saturated hetero-analogs [70]. These derivatives were also tested as potentially antimicrobial compounds (activity against bacteria and fungi *Staphylococcus aureus MRSA*, *Enterococcus faecalis*, *Klebsiella pneumoniae*, *Escherichia coli*, *Salmonella enteritidis*, *Pseudomonas aeruginosa*, *Candida albicans*, *Aspergillus flavus*, *Microsporum gypseum*), but unfortunately all of them showed lower activity than the parent jasmon [71].

Due to the amphiphilicity of the molecule, very good bioavailability through the skin was shown by tetrahydrojasmonic acid—jasmonic acid without double bonds. It is the only jasmonate derivative that is commercially available and used as a cosmetic agent in creams that corrects the appearance of the skin, reduces the appearance of wrinkles and uneven skin tone. In this case, an increase in the expression of hyaluronan synthase 2 and hyaluronan synthase 3 was observed, alongside an increase in hyaluronic acid deposits in the basal and suprabasal layers of the epidermis, stimulation of laminin-5 deposition, collagen IV and fibrillin near the dermal-epidermal junctions (Table 3) [72,73]. The N-terminal conjugate of jasmonic acid with the YPFF-NH_2_ peptide (Table 3) may also have a potentially cosmetic application, but apart from the synthesis, no biological research is yet available [74]. Commercially available acetyl-YPFF tetrapeptide, when applied to the skin, weakens the stimulation of nerve endings, resulting in a reduction in skin hypersensitivity, while jasmonic acid stimulates epidermal renewal. The authors suggest that the peptide derivative of jasmonic acid may reduce the visible effects of aging.

## 8. Conclusions

Jasmonic acid is a natural hormone found in plants. It plays the important role of endogenous regulators of growth and development in plants. The interest in jasmonates and its derivatives occures due to its structural similarity to prostaglandins and shows chance for the potential use of this compound as a therapeutic agent not only in neo-plastic, but also in other diseases. Jasmonates and their derivatives inhibit the proliferation of cancer cells in vitro, such as breast, prostate, melanoma, cervix, colon, colorectal, gastric, hepatoma, lung, myeloid leukemia, neuroblastoma, sarcoma, lymphoblastic leukemia and lymphoma cells. The mechanisms explaining the anti-cancer activity of jasmon compounds proposed so far are based on changes in the ATP level in the cell; induction of re-differentiation through the actions of MAPKs; induction of the apoptosis process via ROS. The development and synthesis of selective jasmonate derivatives have already yielded promising results indicating these compounds in the pharmaceutical products, cosmetic, food industries, and in agriculture.

## Figures and Tables

**Table 1:** Effect of MJ combined with other anticancer agents.

Jasmonates/Drug	Cancer	Concentration Range	Action/Effects	References
**MJ + BCNU** **(in vitro)** **MJ + taxol** **(in vitro)**	Pancreatic cell: PaCa-2 BCL1 MCF-7 DA-3 D-122	MJ: 0.1 mM BCNU: 1, 10, 25 µg/mL-PaCa-2 2.5, 5 µg/mL-BCL1 taxol: 1, 2.5, 5, 10 μg/ml	mitochondriotoxic synergic cytotoxicity IC_50_ ↓	[54]
**MJ + POH** **or** **MJ + cisplatin** **or** **MJ + cisplatin + POH** **(in vitro)**	Breast cancer cell lines: MDA-MB-231 MDA-MB-435 MCF7	IC_2O_ (POH) MDA-MB-231: 0.76 mM MDA-MB-435: 0.6 mM MCF7: 0.8 mM	MJ + POH: cytotoxicity ↑ apoptosis ↑ TNFR1 ↑ MJ + POH: apoptosis ↑	[55]
**MJ + 2DG** **2-deoxyglucose** **(in vitro)**	Sarcoma: SaOS-2 MCA-105	MJ: 0.5–3 mM 2DG: 1 and 2 mM	synergic cytotoxicity ↑ ATP glycolysis ↑	[56]
**MJ + TRAIL** **(in vitro)**	CRC cell lines: SW480, HT29, LS180, HCT116	MJ: 0.5 mM TRAIL: 100–200 ng/ml	IAP (survivin) ↓ caspase activity ↑ TRAIL-induced apoptosis ↑	[58]
**MJ + Smac7N** **(in vitro)**	prostate carcinoma cells: DU145, PC-3 proximal tubular epithelial cells: HK-2	MJ: 0.5–2 mM	Smac7N: MJ-induced cytotoxicity ↑ ran caspase-9 dependent and independent pathways	[60]
**MJ + 3-BrP** **(in vitro)**	Mice breast carcinoma cell line: 4 T1	MJ: 0.5–3 mM 3-BrP: 12.5, 25, 50, 100, 200, 400 μM	ALT ↑ AST ↑ tumor growth ↓ antitumor activity ↑	[53]
**MJ + cisplatin** **MJ + X-rays** **MJ + α-rays**	Cervical cancer cells: SiHa, CaSki, HeLa C33A	MJ: 0.1–1 mM Cisplatin: 0.1–0.5 μM X-rays: 0 25–3 Gy	cell survival ↓ IC_50_ radiation dose ↓ cell viability ↓	[62]

3-BrP—3-bromopyruvate; BCNU—1.3-bis-(2-chloroethyl)-1-nitrosourea; 2DG—2-deoxy-D-glucose; IAP—inhibitors of apoptosis; MJ—methyl Jasmonate; POH—perillyl alcohol; Smac7N—a peptide that contains the N-terminal seven residues of smac; TNFR1—tumor-necrosis factor receptor-1; TRAIL—tumor necrosis factor- (TNF-)related apoptosis-inducing ligand.

**Table 2:** New jasmonate derivatives with anti-cancer activity.

Name and Structure of the Synthetic Derivatives of MJ	Tumor Cells	IC_50_		References
		MJ	Derivative	
**methyl 4,5-didehydrojasmonate DHJM** 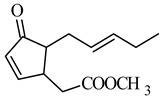	human myeloid leukemia HL-60	347 μM	12 μM	[41]
**methyl 5,7,9,10-tetrabromojasmonate** 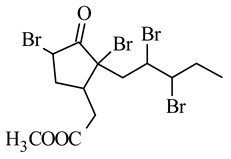	murine melanoma B16-F10	2.6 mM	0.042 mM	[65]
	human lymphoblastic leukemia Molt-4	0.5 mM	0.009 mM	
	human breast cancer MCF7	1.5 mM	0.015 mM	
	human pancreatic cancer MIA PaCa-2	1.4 mM	0.09 mM	
	murine lung cancer D122	1.8 mM	0.25 mM	
**3-((3-methyl-1,2,4-oxadiazol-5-yl) methyl)-2-(pent-2-en-1-yl)cyclo-pentanol** 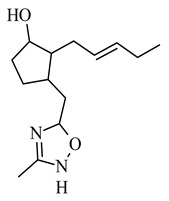	lung carcinoma A549	6.38 mM	4.25 mM	[68]
	ovarian carcinoma SKOV-3	4.17 mM	1.772 mM	

**Table 3:** Synthetic derivatives of jasmonates with biological properties.

Synthetic Derivatives of MJ	Biological Activity	References
**DHJM** 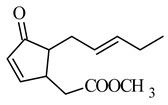	↑ induction of the MAPK activity	[41]
	↑ inhibition in lipopolysaccharide-induced inflammation in the murine macrophages of the RAW264.7 cell line ↑ miRNA-155 in a dose dependent manner ↑ inhibition of the activity of NF-κB-p65 and IκB	[64]
**methyl 5,7,9,10-tetrabromojasmonate** 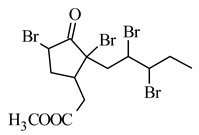	effectively prevents adhesion of B16-F10 cells depending on the dose at sub-toxic concentrations ↑ inhibition of the lung metastases compared to the parent MJ	[65]
**methyl 5-chloro-4,5-didehydrodihydro-jasmonate** 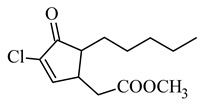	not cytotoxic to RAW 264.7 macrophages ↑ anti-inflammatory activity than natural prostaglandins (PGA1, PGA2 and 15-deoxy-D12,14-PGJ2)	[66]
**t-butyl 5-chloro-4,5-didehydrodihydro-jasmonate** 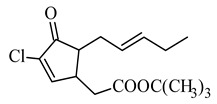	↑ anti-inflammatory activity in vivo than the corresponding chlorinated MJ derivative in the carrageenan paw edema model anti-inflammatory effectiveness comparable to the effect of indomethacin (non-selective COX1 and COX2 inhibitor)	[67]
**3-((3-methyl-1,2,4-oxadiazol-5-yl) methyl)-2-(pent-2-en-1-yl)cyclo-pentanol** 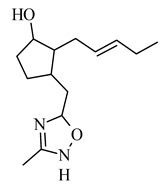	↑ inhibition of enzyme hexokinase II	[68]
**4-methyl-3-(2-pentenyl)-2-oxazolidinone** 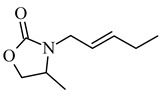	↓ activity against bacteria and fungi *Staphylococcus aureus MRSA, Enterococcus faecalis, Klebsiella pneumoniae, Escherichia coli, Salmonella enteritidis, Pseudomonas aeruginosa, Candida albicans, Aspergillus flavus, Microsporum gypseum* compared to the parent jasmon	[71]
**(3-hydroxy-2-pentylcyclopentyl) acetic acid** 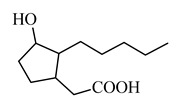	↑ hyaluronan synthase 2 and ↑ hyaluronan synthase 3 ↑ hyaluronic acid deposits in the epidermal layers ↑ deposition of laminin-5, collagen IV and fibrillin near the dermal-epidermal junctions	[72,73]
**JA–YPFF–NH2** 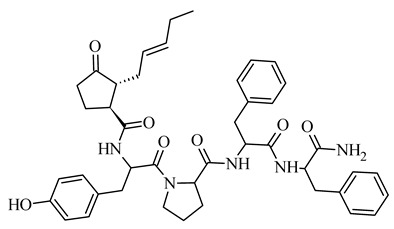	the potential anti-aging agent	[74]
